# Tracking functional recovery in a community-based substance use disorder program: a five-year descriptive evaluation using the brief addiction monitor

**DOI:** 10.1186/s13722-025-00625-3

**Published:** 2025-12-06

**Authors:** Courtney Phillips, Maria C. Mejia, Darian Peters, Jacob Kalathoor, Lea Sacca, Belma Andric

**Affiliations:** 1 Health Care District of Palm Beach, 1515 N. Flagler, Suite 100, West Palm Beach, FL 33401 USA; 2 Schmidt College of Medicine, Florida Atlantic University, Boca Raton, FL USA

**Keywords:** Substance use disorder, Community-based treatment, Brief addiction monitor, Functional recovery, Harm reduction

## Abstract

**Objectives:**

Accessible, evidence-based treatment for substance use disorders (SUDs) remain a public health challenge due to complex clinical and social needs and barriers to long-term engagement. This study describes a five-year evaluation of a low-barrier, outpatient SUD treatment program implemented by the Health Care District of Palm Beach County, Florida, focusing on trends in functional recovery using the Brief Addiction Monitor (BAM) functional assessment.

**Methods:**

Between February 2018 to March 2023, participants with substance use disorders received care through a Federally Qualified Healthcare Center (FQHC) based integrated model offering medication for opioid use disorder, other Medication assisted treatment, behavioral health services, medical , psychiatric, peer services, and care coordination. The BAM was administered at baseline and approximately every three months to assess substance use, risk, and protective factors. Data were analyzed per assessment to reflect variability in patient engagement and follow-up.

**Results:**

A total of 2,425 patients completed 5,277 BAM assessments. Among those with repeated assessments (*n* = 982), the average substance use score declined from 5.19 to 3.45, while the risk score dropped from 14.61 to 11.01. Protective scores increased from 10.65 to 12.40. Reported opiate use decreased from 26.1% at baseline to 17.3% at follow-up. Self-reported overdose history declined from 38.6% to 17.5% (added in 2021). Patient satisfaction improved, with “extremely satisfied” responses rising from 21.6% to 36.5%.

**Conclusions:**

This descriptive evaluation highlights the potential of low-threshold, integrated care models to support recovery-oriented outcomes in real-world settings. Routine use of tools like the BAM enabled multidimensional monitoring of progress despite challenges with retention and data completeness. Findings underscore the importance of flexible, patient-centered approaches to managing the chronic nature of SUD.

**Supplementary Information:**

The online version contains supplementary material available at 10.1186/s13722-025-00625-3.

## Introduction

Substance use disorders (SUDs) represent a persistent public health crisis, imposing significant burdens on healthcare systems, communities, and individuals [1]. Beyond immediate health risks, SUDs are associated with adverse social and economic consequences, including impacts on employment, family stability, and social functioning [1–3]. Despite extensive research and intervention efforts, treatment outcomes remain highly variable, in part due to the chronic nature of SUDs and the complex interplay of biological, psychological, and social determinants influencing recovery [2, 4, 5]. The opioid epidemic has exacerbated this crisis, contributing to a sharp rise in overdose deaths and an unprecedented decline in overall United States (US) life expectancy [6, 7]. Despite efforts to expand access to medications for opioid use disorder (MOUD) and harm reduction initiatives, evaluating long-term recovery outcomes remains complex [8, 9]. Most studies focus on short-term indicators (e.g., retention rates, abstinence at 30 or 90 days, completion of treatment episodes), leaving critical gaps in understanding functional and psychosocial dimensions of recovery that influence sustained well-being and reduced relapse risk as treatment models recognize SUDs as a chronic condition [10–14]. 

A major challenge in evaluating long-term treatment effectiveness is the absence of widely accepted, standardized outcome measures that extend beyond abstinence and program retention [11]. Traditional assessment tools, such as the Addiction Severity Index (ASI) and the Treatment Episode Data Set (TEDS), primarily capture clinical engagement metrics, such as admission and discharge status, rather than functional recovery markers, which are increasingly recognized as critical for sustained well-being [10, 11, 15]. The Brief Addiction Monitor (BAM) is a clinically developed, multidimensional tool with evidence of validity, designed to monitor substance use, associated risk factors, and protective factors over time in individuals receiving treatment for SUD [16]. It has been applied in real-world settings to monitor recovery trajectories beyond abstinence.

Community-based programs are increasingly recognized as effective alternatives to fragmented, episodic care models, offering continuity through federally qualified health centers, harm reduction strategies (e.g., MOUD and overdose prevention), and peer support services bridging emergent and outpatient treatment [13, 17]. The Health Care District of Palm Beach County, Florida, implemented a community-based, integrated SUD treatment model that combines 24/7 emergency addiction care (private hospital) with long-term outpatient management in an federally qualified health centers’ setting and provides these services irrespective of an individual’s insurance coverage or ability to pay. A key feature of this program is the “warm handoff approach”, where peer recovery coaches establish direct connections with patients during acute care episodes and facilitate their transition into long-term outpatient treatment that offers MOUD, psychiatric care, therapy, and primary care and care coordination in a low barrier access approach that is capable of lifelong treatment. Research has demonstrated that warm handoffs significantly improve treatment engagement, reduce stigma-related barriers, and enhance continuity of care [18–20]. 

Nonetheless, real-world programs evaluations remain inherently challenging due to patient attrition, fluctuating engagement, and external barriers (e.g., incarceration, housing instability, and transportation difficulties) [15]. These barriers underscore the importance of standardized functional recovery assessment tools such as BAM to assess long-term treatment effectiveness.

Given the program’s real-world clinical setting, this study takes a descriptive approach to understanding trends in patient-reported outcomes and program implementation challenges over time. Descriptive evaluations of this nature are essential for practice-based research, particularly in community health systems where controlled trials may not be feasible due to variability in patient engagement and care trajectories.

This study presents a five-year descriptive evaluation of a community-based SUD treatment program, using BAM to assess functional recovery beyond abstinence. Specifically, it aims to: (1) describe patterns of BAM implementation and identify challenges in long-term outcome monitoring; and (2) assess trends in substance use, risk factors, and protective factors among patients in a comprehensive SUD treatment program. By providing real-world evidence on functional recovery, the study seeks to inform policy decisions, funding allocations, and future research on standardized long-term outcome measures for addiction treatment.

## Methods

### Design

This study is a descriptive evaluation of a community-based SUD treatment program implemented at a federally qualified health center in South Florida. The evaluation focused on functional recovery outcomes assessed over a five-year period (February 2018–March 2023) using the Brief Addiction Monitor (BAM). The study was approved as exempt research by the Florida Atlantic University Institutional Review Board (IRB). All data were fully de-identified and managed according to ethical standards for human subjects research.

### Implementation of BAM and program evaluation considerations

BAM implementation was examined by reviewing data collection methods, administration patterns, and consistency across a real-world clinical setting. Feasibility was considered in terms of BAM administration rates, repeat completion, and adaptability to changing clinical workflow. Due to electronic medical record (EMR) transitions and staffing variability, BAM was administered in multiple formats (clinician-led, tablet-based, or hybrid). In 2021, a non-validated overdose history question was added to enhance clinical risk tracking. Implementation challenges included staffing shifts, resource constraints, and disruptions from the COVID-19 pandemic, which affected BAM completion consistency. To contextualize BAM completion trends, patient re-admission patterns and the number of assessments completed at key time intervals (e.g., 3, 6, 12 months) were analyzed and are presented in Table 1 and Appendix Table A1.

**Table 1:** Participant characteristics and program engagement

Variables	Frequency (*N*)	Percentage (%)
**Sex (*n*** **=** **2**,**425)**		
Male	1,632	67.30
Female	793	32.70
**Ethnicity (*n*** **=** **2**,**425)**		
Hispanic	319	13.15
Not Hispanic or Latino	1,906	78.60
Unreported	200	8.25
**Race (*n*** **=** **2**,**425)**		
White	1,808	74.56
Black or African American	261	10.76
More than 1 Race	104	4.29
Other Race	62	2.56
American Indian or Alaska Native	15	0.62
Asian	3	0.12
Native Hawaiian	1	0.04
Pacific Islander	4	0.16
Unreported	167	6.89
**Number of Re-admissions (*n*** **=** **2**,**425)**		
0	1,443	59.51
1	400	16.49
2	168	6.93
3	137	5.65
4	86	3.55
5	63	2.60
6	38	1.57
7	24	0.99
8	20	0.82
9	12	0.49
10	10	0.41
11	11	0.45
12	9	0.37
13	3	0.12
14	1	0.04

### Setting and participants

The evaluation was conducted at a federally qualified health center operated by the Health Care District of Palm Beach County, Florida. The center provides integrated primary care and behavioral health services, including medication for opioid use disorder (MOUD), psychiatric care, group and individual counseling, peer support, pharmacy services, and care coordination. All individuals seeking treatment for substance use disorders (SUDs) were eligible to participate regardless of treatment history, motivation level, insurance status, or ability to pay. Participants entered the program through multiple referral pathways, including self-referrals (walk-ins), hospital transfers from the Emergency Room Addiction Stabilization Unit with a peer support specialist doing a warm handoff, and referrals from the Health Care District primary care providers, community organizations, child welfare agencies, and the criminal justice system. A countywide public awareness campaign helped increase access by promoting the program’s inclusive and low-barrier model.

### Program description

The comprehensive SUD treatment model integrated acute stabilization, warm handoffs to outpatient care, and long-term treatment within a single system. Patients presenting in acute crisis received 24/7 emergency addiction care at a private clinic emergency department. Following stabilization, they were linked to outpatient services at the federally qualified health center. Warm handoffs were facilitated by peer recovery coaches, who engaged patients during emergency episodes and accompanied them through the transition to outpatient care. These peers provided ongoing support regardless of a patient’s readiness to change, fostering trust and engagement throughout the recovery process.

The outpatient program offered same-day access to evidence-based MOUD (e.g., buprenorphine-naloxone), psychiatric evaluation, individual and group counseling, peer support, pharmacy services, and case management. Patients were not discharged for relapse or nonadherence, and appointments were scheduled without delay, reinforcing the program’s low-barrier, harm-reduction approach. Individuals were welcome at any stage of motivation, including those uncertain about abstinence or who had previously disengaged from care. Upon enrollment, patients provided informed consent for clinical assessment and program evaluation. They also agreed to program policies and regulatory guidelines, including those related to controlled substances and buprenorphine-naloxone protocols.

### Data collection

The Brief Addiction Monitor, Version 2.0, is a 17-item clinical assessment tool used to monitor treatment progress in individuals with substance use disorders. It includes items assessing substance use (Questions 4–6), risk factors (Questions 1–3, 8, 11, 15), and protective factors (Questions 9–10, 12–14, 16) [16]. The BAM was administered at baseline and approximately every 90-day thereafter, though actual intervals varied based on staffing capacity and workflow constraints. Due to inconsistent recall and documentation, responses to Question 7 (type and frequency of drug use) were analyzed as a binary variable (yes/no), rather than using the original categorical frequency scale (e.g., 1–5). This approach enabled consistent coding across records while limiting potential misclassification. An additional self-reported overdose question (non-validated) was added in 2021 to assess emergency service utilization risk.

Data were extracted from the EMR, anonymized, and stored securely. Patients completed between 1 and 15 BAM assessments, depending on their engagement duration. Due to limitations in unique patient identifiers, not all individuals could be matched to a baseline BAM. Some patients re-entered care and completed a new BAM at a follow-up interval (e.g., 3 months), which was treated as an initial assessment in the dataset. A per-assessment analysis approach was used to reflect the variability in follow-up intervals and treatment episodes.

### Data analysis

Patient data were extracted from the electronic-medical-record (EMR), then anonymized, and stored in a securely. Due to wide variability in follow-up timing and treatment duration, we used a per-assessment analysis approach rather than a fixed-interval analysis. This allowed us to include all available BAM assessments and better reflect the episodic nature of care engagement in real-world SUD treatment settings. If a patient left the program and re-enrolled at a later time, a new BAM baseline was recorded and treated as a separate episode of care. To account for these instances, we reported outcomes by the total number of BAMs completed rather than tracking calendar time from initial enrollment. While this method limits direct causal inference, it accommodates fluctuating engagement and high turnover, which are common challenges in community-based SUD programs.

The primary outcome was functional recovery, assessed through changes in BAM scores over successive assessments across three domains: (1) substance use score (sum of Questions 4–6, range 0–12) measures the frequency of alcohol and illicit drug use over the past 30 days; (2) risk score (sum of Questions 1–3, 8, 11, 15, range 0–24) assesses vulnerability factors associated with continued substance use, including physical health, emotional distress, and housing instability; and (3) protective factors score (sum of Questions 9–10, 12–14, 16, range 0–24) evaluates recovery supports such as self-efficacy, social connectedness, and financial stability. Secondary outcomes included responses to: self-reported drug use (Question 7), which was dichotomized (any reported use: yes/no) due to inconsistent recall was and response patterns; overdose history (Question 18, “How many overdoses have you had in the last three months?” categorized as 0, 1–2, 3–5, or > 5); and patient satisfaction with recovery progress (Question 17, five-point Likert scale).

Descriptive statistics were used to examine score trajectories across repeated assessments. We stratified summary trends by the number of BAMs completed (e.g., 1, 2, 3 + assessments) to partially address concerns around attrition bias and recovery trajectory. Table 1 and Appendix Table A1 provide additional detail on the number and timing of BAMs completed. Trendlines in the figures are presented solely for illustrative purposes to show overall directionality and were not generated using formal regression models.

Given the observational nature of the evaluation, limited covariate data, and inconsistent follow-up intervals, we did not conduct inferential statistical modeling, such as linear mixed effects or regression analysis. This was a deliberate decision in line with the study’s descriptive goals. However, findings may serve as preliminary evidence to guide more rigorous designs in future work.

## Results

### Sample characteristics and BAM completion patterns

A total of 2,425 unique patients completed at least one Brief Addiction Monitor (BAM) assessment between February 2018 and March 2023, resulting in 5,277 completed assessments. Of these, 1,443 (59.5%) completed only one assessment, while 982 (40.5%) completed two or more. Among those with follow-up assessments, 568 patients (23.4%) completed three or more. The number of BAM assessments per patient ranged from 1 to 15, with an average of 2.2 (SD = 2.3) and a median of 2. Demographic characteristics are presented in Table 1. The majority of participants were male (67.3%) and identified as White (74.6%), while 13.2% identified as Hispanic or Latino.

Patterns of BAM completion over time are detailed in Appendix Table A1. Note that Table A1 reflects counts of BAM assessments by time interval and not unique patients; thus, totals differ from the patient-level frequencies described above.

### BAM administration trends and challenges

BAM administration format varied throughout the study period due to transitions in electronic medical records (EMR), staffing fluctuations, and workflow adjustments. The instrument was initially clinician-administered, later shifted to tablet-based self-reporting, and eventually returned to clinician-led administration following an EMR change. These operational changes, combined with disruptions caused by the COVID-19 pandemic, contributed to inconsistencies in follow-up assessments and high rates of attrition. However, due to limitations in the program’s EHR infrastructure, we were unable to determine whether missed follow-up assessments were due to patients disengaging from care, changes in administration workflows, or other external factors. As such, missing follow-up data should be interpreted with caution, and we cannot attribute non-completion to any single cause.

### Feasibility of BAM administration

Feasibility was assessed based on three practical indicators: successful administration, frequency of repeated assessments, and data retrievability from the EMR. Over the five-year study period, the BAM was implemented in routine clinical workflows at a community-based outpatient SUD program. Of the 2,425 patients who completed at least one BAM, 40.5% (*n* = 982) received at least one follow-up assessment, and 23.4% (*n* = 568) completed three or more assessments. While only a subset of patients reached these thresholds, the ability to sustain repeated administrations over extended periods demonstrates partial feasibility in a real-world setting. BAM assessments were retrievable through the EMR for analysis, further supporting the viability of its use in ongoing program evaluation. However, challenges related to staffing, electronic record transitions, and patient engagement affected consistency in administration.

### Trends in functional recovery

**Fig. 1:**
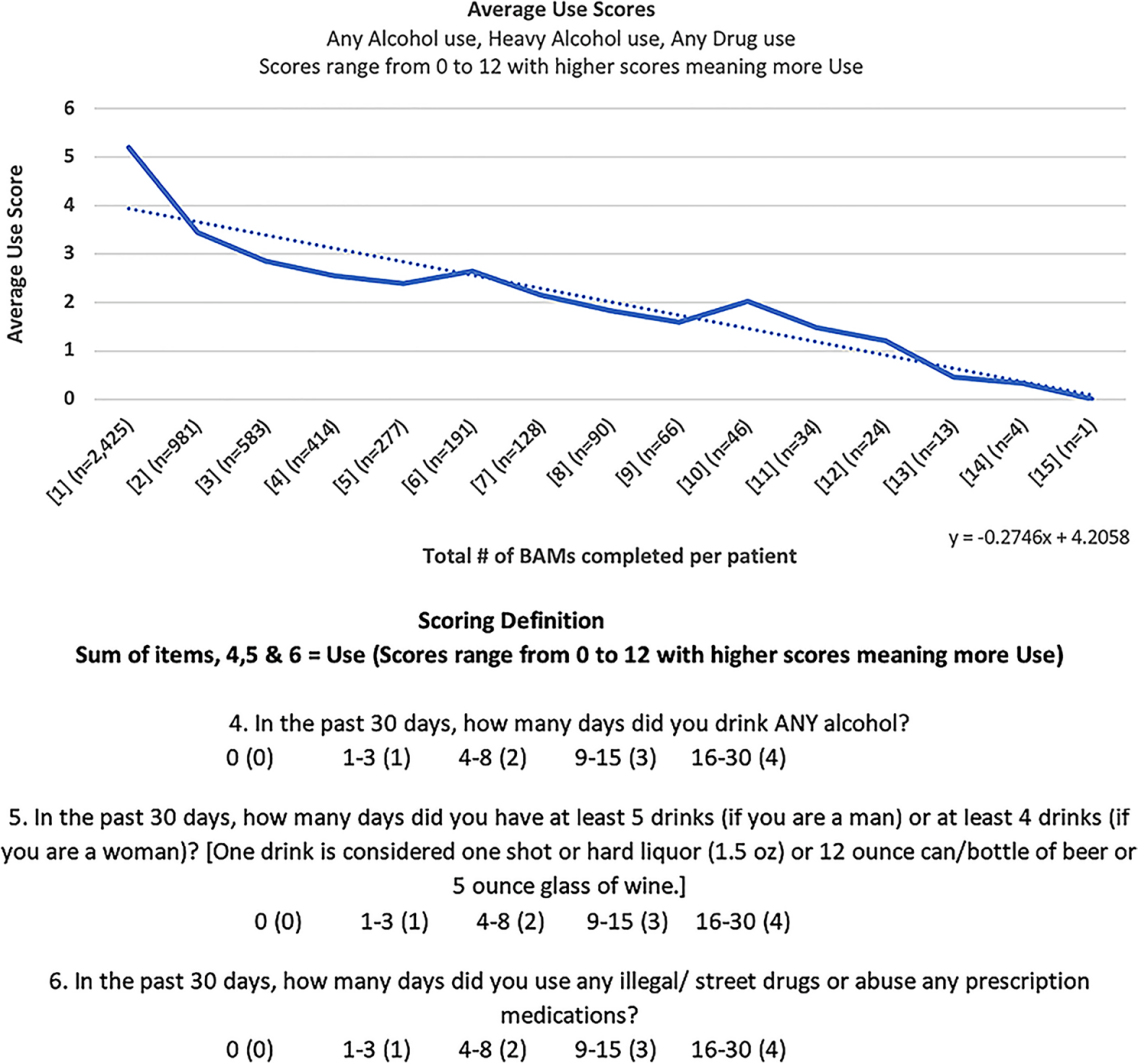
Average Brief Addiction Monitor Substance-Use Scores Across Successive Assessments. Line (or bar) plot depicting the mean substance-use score (range 0–12) at baseline (Assessment 1) and at each subsequent 3-month BAM assessment (Assessments 2–6). Lower scores indicate reduced frequency of alcohol or illicit-drug use in the past 30 days. Error bars represent ± 1 standard deviation. Trendlines shown are descriptive and not based on inferential statistical modeling

Among patients with at least two BAM assessments, improvements were observed across all three BAM domains. The mean substance use score decreased from 5.19 at the first assessment to 3.45 by the second, with continued decline noted in subsequent assessments. This trend suggests a reduction in recent alcohol and illicit drug use over time (Fig. 1).

**Fig. 2:**
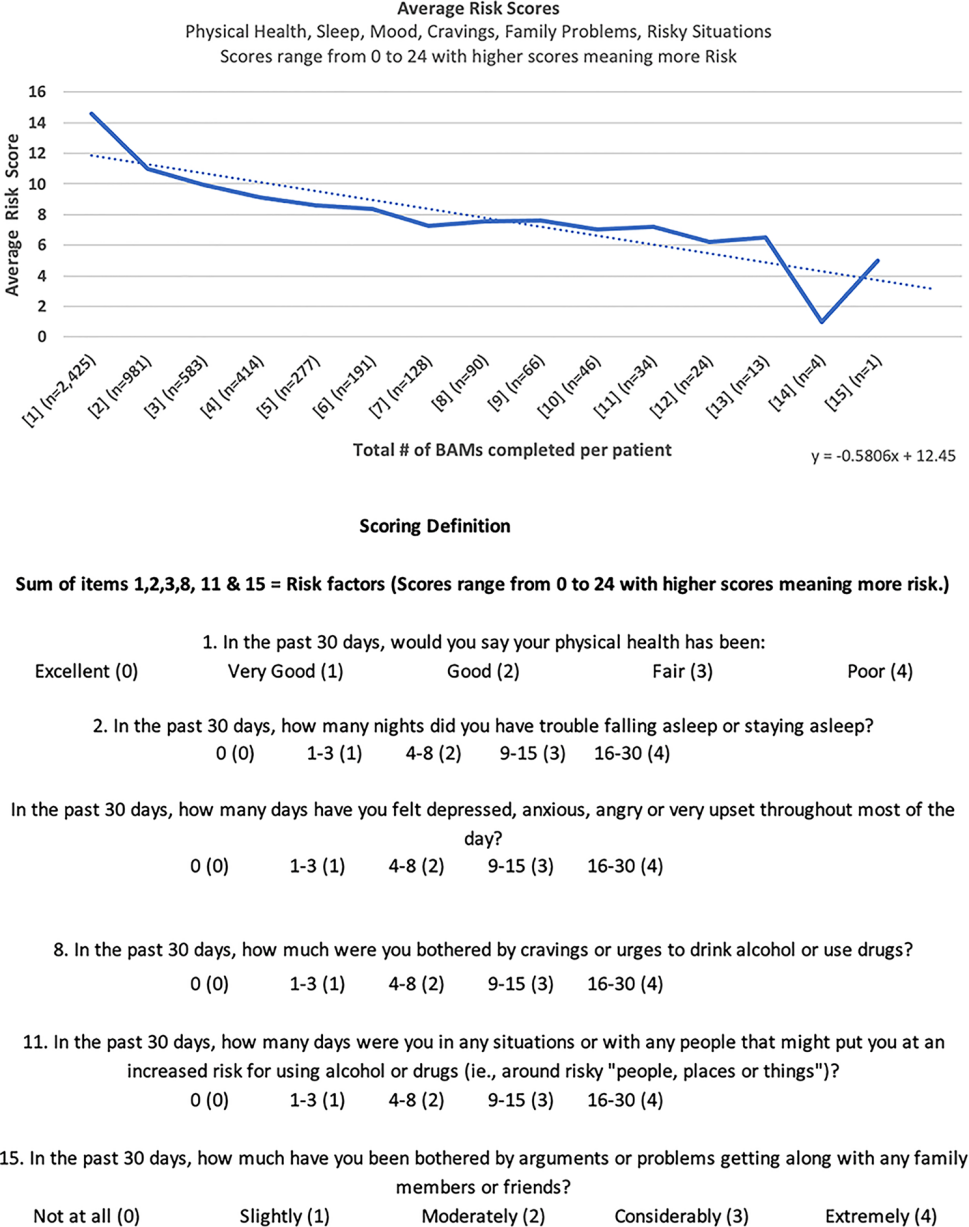
Average Brief Addiction Monitor Risk Scores Across Successive Assessments. Line (or bar) plot showing the mean risk score (range 0–24) at baseline (Assessment 1) and at each subsequent 3-month BAM assessment (Assessments 2–6). Lower scores indicate reduced physical, psychological, and social risk factors associated with substance use. Error bars denote ± 1 standard deviation. Trendlines shown are descriptive and not based on inferential statistical modeling

**Fig. 3:**
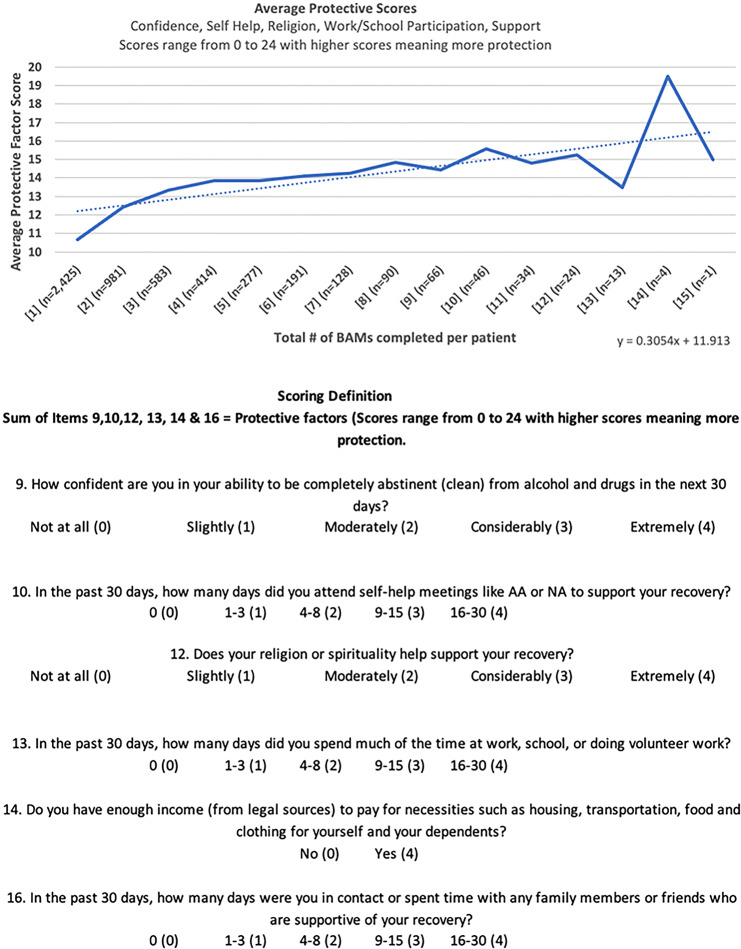
Average Brief Addiction Monitor Protective Scores Across Successive Assessments. Line (or bar) plot illustrating the mean protective score (range 0–24) at baseline (Assessment 1) and at each subsequent 3-month BAM assessment (Assessments 2–6). Higher scores reflect stronger protective factors such as self-efficacy, social support, and financial stability. Error bars represent ± 1 standard deviation. Trendlines shown are descriptive and not based on inferential statistical modeling

Risk scores, which reflect physical and emotional vulnerability, declined from a baseline mean of 14.61 to 11.01 at the second assessment, with further decreases across later timepoints (Fig. 2). Similarly, protective scores, which assess recovery supports such as self-efficacy and social connectedness, increased from an average of 10.65 at baseline to 12.40 at the second assessment, with continued improvement observed thereafter (Fig. 3).

### Self-reported drug use and overdose history

**Fig. 4:**
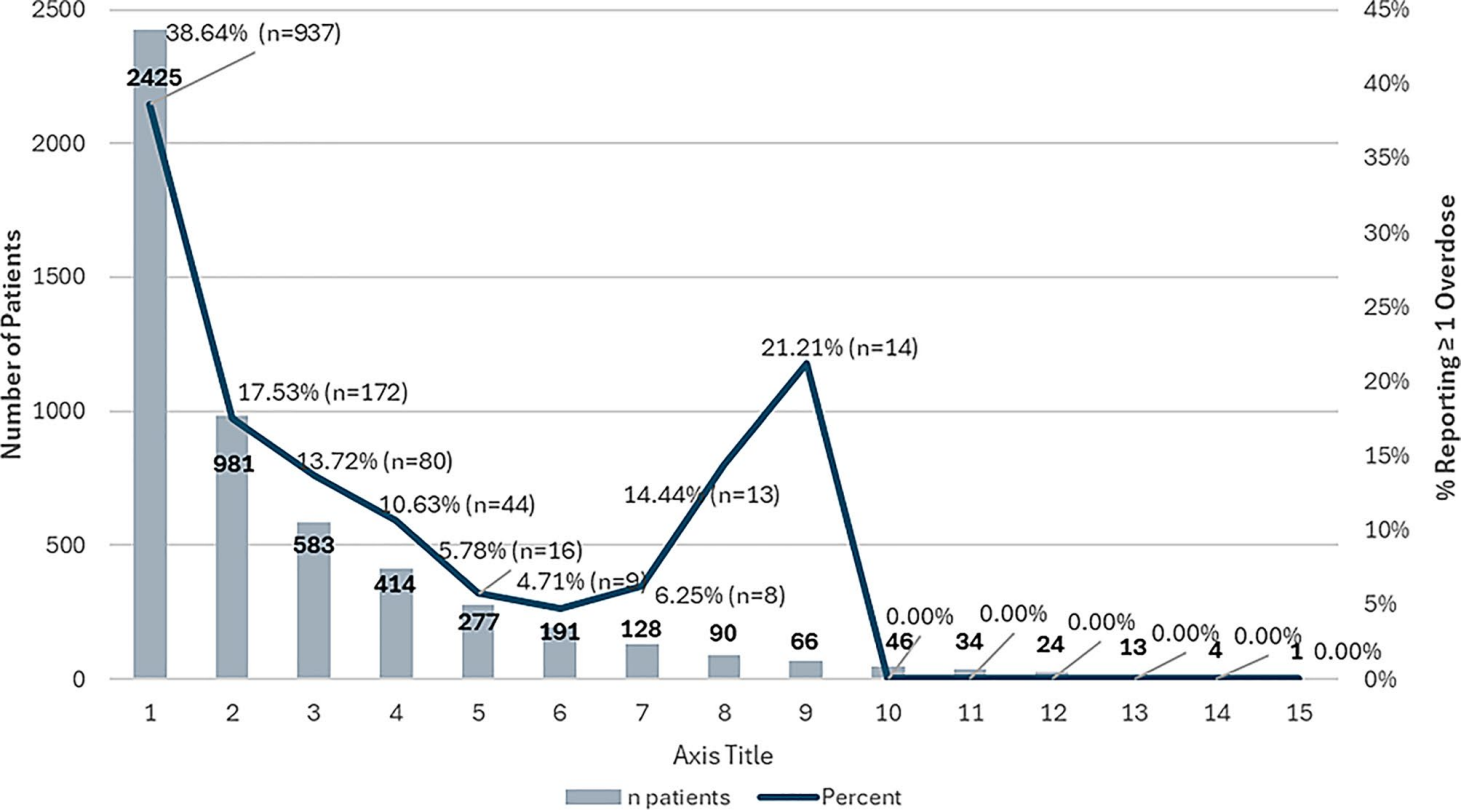
Self-Reported Overdose Events in the Past 3 Months. Bar chart displaying the percentage of patients reporting at least one overdose within the past 3 months at baseline (Assessment 1) and at the next completed BAM assessment (Assessment 2). The marked decline illustrates reduced overdose incidence following program engagement. Trendlines shown are descriptive and not based on inferential statistical modeling

Question 7, which captures the number of days of specific substance use in the past 30 days, was analyzed in binary format due to inconsistencies in recall. Between the first and second assessments, reductions were observed in reported use of several substances. For example, marijuana use decreased from 25.1% to 22.5%, opiate use from 26.1% to 17.3%, sedatives/tranquilizers from 14.85% to 9.28%, and crack/cocaine use from 13.5% to 8.4%. Likewise, reported use of other substances (e.g., steroids, non-prescription sleep aids, Benadryl) declined from 1.24% to 0.31%. This proportion declined to 17.5% at the second assessment and continued to decrease through subsequent assessments, suggesting possible reductions in high-risk use among those who remained engaged in care. However, a modest increase was observed at BAMs 9 and 10, which may reflect small sample sizes or selective follow-up of high-risk individuals (Fig. 4).

### Patient satisfaction

Patient satisfaction with treatment progress, measured by BAM Question 17, improved over time. At baseline, 21.6% of patients reported being “extremely satisfied” with their recovery, which increased to 36.5% by the second BAM. Conversely, the proportion reporting they were “not at all satisfied” decreased from 8.8% to 5.2%.

## Discussion

This descriptive evaluation of a community-based SUD program demonstrates how integrated, low-barrier care models can support functional recovery in real-world, safety-net settings. Among patients who completed multiple BAM assessments, we observed consistent improvements in substance use reduction, risk factor mitigation, and enhancement of protective factors. These changes were accompanied by increased satisfaction with treatment progress and reduced self-reported overdose history over time. While the observational nature of the data limits causal inference, these findings offer valuable insights into patient trajectories and the potential role of routine outcome monitoring in community-based care.

The BAM was adaptable to our setting despite significant implementation challenges including staff turnover, EMR transitions, and the COVID-19 pandemic [21–24]. Repeated administration allowed for multi-dimensional tracking of recovery progress across substance use behaviors, health and social risk factors, and resilience-building supports. These patterns reinforce BAM’s utility in clinical settings where patient engagement may be intermittent, particularly for programs serving individuals with co-occurring conditions, housing instability, or criminal justice involvement [21–24].

While BAM implementation was feasible, several challenges limited consistency across time points and patients. Fewer than half of patients received two or more BAM assessments, and less than a quarter received three or more. These rates, though modest, demonstrate potential feasibility in real-world conditions marked by staffing variability, EMR transitions, and the COVID-19 pandemic. The successful integration of BAM into the EMR and use of its data for longitudinal tracking also support feasibility. However, the lack of standardized administration protocols and variable engagement duration reduced internal validity and hindered the ability to make comparisons over time. These findings suggest that with adequate infrastructure and dedicated workflows, BAM could be implemented more consistently in similar safety-net care settings.

Most participants completed only one BAM, and fewer than a quarter completed three or more assessments. High attrition and variable follow-up reflect the challenges of maintaining engagement in outpatient care for individuals with complex needs [21, 24, 25]. Reasons for disengagement such as incarceration, loss to follow-up, or systemic barriers, were not captured and remain a limitation. However, even limited BAM data provided meaningful insights into recovery patterns. Our use of a per-assessment analytic approach accommodated irregular follow-up intervals and allowed us to summarize trends across the full sample.

This approach aligns with recovery-oriented models that view recovery as a non-linear, individualized process rather than a binary outcome [12]. The observed improvements in self-efficacy, peer support, and emotional stability are particularly notable given that many participants were not initially seeking abstinence-based care. Peer recovery coaches played a key role in engaging patients at crisis points and facilitating warm handoffs to outpatient services, an approach supported by evidence in similar settings [18, 20]. The addition of a non-validated overdose history item in 2021 provided a clinically useful but unstandardized measure of high-risk use. Declines in reported overdoses among patients completing follow-up BAMs suggest potential benefit, though further validation of this item is needed [25]. The observed uptick in overdose reports at BAMs 9 and 10 warrants cautious interpretation. Given the small number of patients completing these later assessments, this increase may reflect sampling variability or selective retention of higher-risk individuals who remained in care. These patients may represent a subgroup with more complex needs or repeated episodes of relapse, underscoring the importance of sustained monitoring and individualized support over time.

This evaluation has several limitations. It was based on real-world EMR data with variable completeness, no control group, and limited availability of covariates. Social desirability bias may have affected self-reported responses [21, 26]. We were unable tostratify results by race, gender, or other sociodemographic factors, despite known disparities in care engagement and outcomes have been documented among Hispanic/Latino populations and women with SUD [27–30]. Inconsistent BAM administration across patients and time points limits both generalizability and internal validity. This inconsistency stemmed from workflow disruptions, staffing variability, EMR transitions, and the COVID-19 pandemic. Additionally, the BAM was not always administered at consistent intervals, and some patients lacked a clearly defined baseline assessment, making it difficult to track individualized recovery trajectories. These factors may have introduced measurement bias, limited the representativeness of longitudinal findings, and resulted in non-random patterns of missing data.

Finally, while our findings suggest potential value in using the BAM as a functional outcome tool in community-based SUD care, we caution against causal interpretation. In addition to the observational nature of the dataset, we did not apply inferential statistical models (e.g., mixed-effects or generalized estimating equations) to adjust for confounding or account for repeated measures. Our trend analyses are descriptive and exploratory in nature. These methodological constraints underscore the need for prospective, controlled studies to rigorously evaluate BAM’s impact and implementation in diverse settings.

Despite these constraints, the findings highlight key lessons for practice and policy. Low-threshold SUD treatment models that integrate MOUD, behavioral health, and peer services may promote engagement even among patients with low initial readiness for change [22, 23]. Embedding outcome monitoring tools like BAM into routine care enable teams to track patient progress, guide care coordination, and justify continued investment in integrated services. As Florida’s Coordinated Opioid Recovery (CORe) network expands BAM use statewide insights from this evaluation may help inform broader implementation strategies [25]. 

## Conclusions

This evaluation of a low-barrier, community-based SUD treatment program illustrates how integrated care models can support functional recovery among individuals with complex needs, even in the absence of rigid adherence requirements. While most patients completed only one BAM assessment, those who remained engaged demonstrated measurable improvements in substance use, risk reduction, and recovery supports. These findings underscore the value of incorporating routine, multidimensional outcome monitoring into real-world clinical settings. Future research should further explore implementation strategies to improve retention and expand the validity of emerging tools, including non-validated overdose tracking measures. As states and systems expand efforts to scale integrated SUD care, evaluations rooted in real-world data can help guide policy, enhance quality improvement, and center recovery as a process and not a threshold.

## Supplementary Information

Below is the link to the electronic supplementary material.

Supplementary Material 1

Supplementary Material 2

## Data Availability

The data that support the findings of this study are not openly available due to reasons of sensitivity and are available from the corresponding author upon reasonable request.

## References

[1] Robinson SM, Adinoff B. The classification of substance use disorders: Historical, Contextual, and conceptual considerations. Behav Sci (Basel). 2016;6(3):18. 10.3390/bs6030018. PMID: 27548233; PMCID: PMC5039518. 27548233 10.3390/bs6030018 PMC5039518

[2] Volkow ND, Blanco C. The changing opioid crisis: development, challenges and opportunities. Mol Psychiatry. 2021;26(1):218–33. 10.1038/s41380-020-0661-4. Epub 2020 Feb 4. PMID: 32020048; PMCID: PMC7398847. 32020048 10.1038/s41380-020-0661-4 PMC7398847

[3] National Institute on Drug Abuse. Substance use in women: sex and gender differences in substance use. 2022. Available at: https://nida.nih.gov/publications/research-reports/substance-use-in-women.

[4] Jalali MS, Botticelli M, Hwang RC, Koh HK, McHugh RK. The opioid crisis: a contextual, social-ecological framework. Health Res Policy Syst [Internet]. 2020;18(1):87. Available from: 10.1186/s12961-020-00596-8. 10.1186/s12961-020-00596-8 PMC7409444 32762700

[5] Kelly JF, Bergman B, Hoeppner BB, Vilsaint C, White WL. Prevalence and pathways of recovery from drug and alcohol problems in the united States population: implications for practice, research, and policy. Drug Alcohol Depend. 2017;181:162–9. 10.1016/j.drugalcdep.2017.09.028. Epub 2017 Oct 18. PMID: 29055821; PMCID: PMC6076174. 29055821 10.1016/j.drugalcdep.2017.09.028 PMC6076174

[6] Spencer MR, Garnett MF, Miniño AM. Drug overdose deaths in the United Sates, 2002–2022. Hyattsville, MD: National Center for Health Statistics; 2024. NCHS Data Brief, no 491.

[7] Hedegaard H, Minino AM, Warner M. Drug overdose deaths in the United States, 1999–2021. Hyattsville, MD: National Center for Health Statistics; 2023. NCHS Data Brief, no 457.

[8] McKay JR, Impact of Continuing Care on Recovery From Substance Use Disorder. Alcohol Res. 2021;41(1):01. 10.35946/arcr.v41.1.01. Published 2021 Jan 21. 33500871 10.35946/arcr.v41.1.01 PMC7813220

[9] National Academies of Sciences, Engineering, and Medicine. Medications for opioid use disorder save lives. Washington, DC: National Academies; 2019. 10.17226/25310. 30896911

[10] Garnick D, Horgan C, Mark TL, et al. The importance of identification when measuring performance in addiction treatment. Subst Abus. 2019;40(3):263–7. 10.1080/08897077.2019.1580240. 30913002 10.1080/08897077.2019.1580240 PMC6763371

[11] Tiffany ST, Friedman L, Greenfield SF, Hasin DS, Jackson R. Beyond drug use: a systematic consideration of other outcomes in evaluations of treatments for substance use disorders. Addiction. 2012;107(4):709–18. 10.1111/j.1360-0443.2011.03581.x. Epub 2011 Oct 7. PMID: 21981638; PMCID: PMC3257402. 21981638 10.1111/j.1360-0443.2011.03581.x PMC3257402

[12] Blanco C, Wiley TRA, Lloyd JJ, Lopez MF, Volkow ND. America’s opioid crisis: the need for an integrated public health approach. Translational Psychiatry. 2020;10(1):167. 10.1038/s41398-020-0847-1. 32522999 10.1038/s41398-020-0847-1 PMC7286889

[13] Paquette CE, Daughters SB, Witkiewitz K. Expanding the continuum of substance use disorder treatment: nonabstinence approaches. Clin Psychol Rev. 2022;91:102110. 10.1016/j.cpr.2021.102110. 34864497 10.1016/j.cpr.2021.102110 PMC8815796

[14] McLellan AT, Lewis DC, O’Brien CP, Kleber HD. Drug dependence, a chronic medical illness: implications for treatment, insurance, and outcomes evaluation. JAMA. 2000;284(13):1689-95. 10.1001/jama.284.13.1689. PMID: 11015800. 10.1001/jama.284.13.1689 11015800

[15] Scott CK, Dennis ML, Grella CE, Watson DP, Davis JP, Hart MK. Using recovery management checkups for primary care to improve linkage to alcohol and other drug use treatment: a randomized controlled trial three month findings. Addiction. 2023;118(3):520–32. 10.1111/add.16064. Epub 2022 Oct 29. PMID: 36208061; PMCID: PMC10015976. 36208061 10.1111/add.16064 PMC10015976

[16] Cacciola JS, Alterman AI, Dephilippis D, Drapkin ML, Valadez C Jr, Fala NC, Oslin D, McKay JR. Development and initial evaluation of the brief addiction monitor (BAM). J Subst Abuse Treat. 2013;44(3):256–63. 10.1016/j.jsat.2012.07.013. Epub 2012 Aug 14. PMID: 22898042; PMCID: PMC3602977. 22898042 10.1016/j.jsat.2012.07.013 PMC3602977

[17] Langabeer JR 2nd, Yatsco A, Champagne-Langabeer T. Using telehealth to address the opioid epidemic in rural areas: an integrative review. BMC Health Serv Res. 2021;21(1):1–11.

[18] Krawczyk N, Fawole A, Yang J, Tofighi B. Early innovations in opioid use disorder treatment and harm reduction during the COVID-19 pandemic: a scoping review. Addict Sci Clin Pract [Internet]. 2021 [cited 2023 Feb 9];16(1):68. Available from: https://pubmed.ncbi.nlm.nih.gov/34774106/.. 10.1186/s13722-021-00275-1 PMC8590133 34774106

[19] Anand P, Desai N. Correlation of warm handoffs versus electronic referrals and engagement with mental health services co-located in a pediatric primary care clinic. J Adolesc health: official publication Soc Adolesc Med. 2023;73(2):325–30. 10.1016/j.jadohealth.2023.02.032. 10.1016/j.jadohealth.2023.02.032 37061906

[20] Jack HE, Oller D, Kelly J, Magidson JF, Wakeman SE. Addressing substance use disorder in primary care: the role, integration, and impact of recovery coaches. Subst Abuse. 2018;39(3):307–14. 10.1080/08897077.2018.1485135. 10.1080/08897077.2017.1389802 28991516

[21] Latkin CA, Edwards C, Davey-Rothwell MA, Tobin KE. The relationship between social desirability bias and self-reports of health, substance use, and social network factors among urban substance users in Baltimore, Maryland. Addict Behav. 2017;73:133–6. 10.1016/j.addbeh.2017.05.005. 28511097 10.1016/j.addbeh.2017.05.005 PMC5519338

[22] Burlew AK, Copeland VC, Ahuama-Jonas C, Calsyn DA. Does cultural adaptation have a role in substance abuse treatment? Soc Work Public Health. 2013;28(3–4):440–60. 10.1080/19371918.2013.774811. 23731430 10.1080/19371918.2013.774811 PMC4220306

[23] Mejia MC, Kowalchuk A, Gonzalez S, Sunny A, Scamp N. Expanding Treatment, Recovery, and reentry services for female offenders: improving outcomes through Client-Centered interventions. Community Ment Health J. 2024;60(4):713–21. 10.1007/s10597-023-01223-w. Epub 2024 Jan 4. PMID: 38175318. 38175318 10.1007/s10597-023-01223-w

[24] Krawczyk N, Negron T, Nieto M, Agus D, Fingerhood MI. Overcoming medication stigma in peer recovery: A new paradigm. Subst Abus. 2018;39(4):404–9. 10.1080/08897077.2018.1439798. Epub 2018 Apr 4. PMID: 29432086; PMCID: PMC6087684. 29432086 10.1080/08897077.2018.1439798 PMC6087684

[25] Markatou M, Kennedy O, Brachmann M, Mukhopadhyay R, Dharia A, Talal AH. Social determinants of health derived from people with opioid use disorder: improving data collection, integration and use with cross-domain collaboration and reproducible, data-centric, notebook-style workflows. Front Med. 2023;10:1076794. 10.3389/fmed.2023.1076794. 10.3389/fmed.2023.1076794 PMC10017859 36936205

[26] Mejia MC, Kowalchuk A, Gonzalez SJ, Nair M, Webb L, Scamp N. Challenges and implications for substance use and mental healthcare among under-resourced women in the COVID-19 Era. Cureus. 2024;16(6):e62452. PMID: 39015873. 10.7759/cureus.62452. 10.7759/cureus.62452 PMC11250514 39015873

[27] U.S. Census Bureau quickfacts: West Palm Beach City, Florida. United States Census Bureau. Accessed February 14. 2025. https://www.census.gov/quickfacts/fact/table/westpalmbeachcityflorida/POP645222.

[28] Hai AH, Lee CS, Abbas BT, Bo A, Morgan H, Delva J. Culturally adapted evidence-based treatments for adults with substance use problems: A systematic review and meta-analysis. Drug Alcohol Depend. 2021;226:108856. 10.1016/j.drugalcdep.2021.108856. Epub 2021 Jun 24. PMID: 34274617; PMCID: PMC11468295. 34274617 10.1016/j.drugalcdep.2021.108856 PMC11468295

[29] Barnett ER, Knight E, Herman RJ, Amarakaran K, Jankowski MK. Difficult binds: A systematic review of facilitators and barriers to treatment among mothers with substance use disorders. J Subst Abuse Treat. 2021;126:108341. 10.1016/j.jsat.2021.108341. Epub 2021 Feb 27. PMID: 34116826. 34116826 10.1016/j.jsat.2021.108341

[30] Caetano T, Pinho MS, Ramadas E, Lopes J, Areosa T, Ferreira D, et al. Substance abuse and susceptibility to false memory formation: a systematic review and meta-analysis. Front Psychol. 2023;14. 10.3389/fpsyg.2023.1176564. 10.3389/fpsyg.2023.1176564 PMC10196796 37213356

