# Supplementary material for: Tracking functional recovery in a community-based substance use disorder program: a five-year descriptive evaluation using the brief addiction monitor
Source: Addict Sci Clin Pract. 2025 Dec 6;21:4. doi: 10.1186/s13722-025-00625-3 (PMC12805790; doi:10.1186/s13722-025-00625-3)
Supplement: Supplementary file 1 — Supplementary Material 1 [file 13722_2025_625_MOESM1_ESM.docx]

| **Domain** | **Item #** | **Question** | **Response Type** | **Scoring Use** |
| --- | --- | --- | --- | --- |
| **Substance Use** | 4 | Days used any alcohol in the past 30 days | 0–30 days | Substance Use Score |
|  | 5 | Days had ≥5 drinks (≥4 if female or age >65) in past 30 days | 0–30 days | Substance Use Score |
|  | 6 | Days used illegal drugs or misused prescription medications in past 30 days | 0–30 days | Substance Use Score |
| **Risk Factors** | 1 | Physical health over the past 30 days | 5-point scale (Excellent–Poor) | Risk Score |
|  | 2 | Nights with sleep problems in past 30 days | 0–30 nights | Risk Score |
|  | 3 | Days felt depressed, anxious, angry, or upset in past 30 days | 0–30 days | Risk Score |
|  | 8 | How much bothered by cravings in past 30 days | 5-point scale (Not at all–Extremely) | Risk Score |
|  | 11 | Days exposed to high-risk people/places in past 30 days | 0–30 days | Risk Score |
|  | 15 | How much bothered by family/friend conflict | 5-point scale (Not at all–Extremely) | Risk Score |
| **Protective Factors** | 9 | Confidence in ability to remain abstinent in next 30 days | 5-point scale (Not at all–Extremely) | Protective Score |
|  | 10 | Days attended support groups (AA, NA, etc.) in past 30 days | 0–30 days | Protective Score |
|  | 12 | Does religion/spirituality support recovery? | Yes / No | Protective Score |
|  | 13 | Days worked, studied, or volunteered in past 30 days | 0–30 days | Protective Score |
|  | 14 | Have sufficient income to meet basic needs? | Yes / No | Protective Score |
|  | 16 | Days spent with recovery-supportive family/friends in past 30 days | 0–30 days | Protective Score |
| **Other Items** | 7 | Days used specific substances in past 30 days (marijuana, stimulants, opioids, etc.) – Sub-items 7A–7G | 0–30 days (per substance) | Descriptive Only (not scored) |
|  | 17 | Satisfaction with progress toward recovery goals | 5-point Likert scale | Secondary Outcome |
|  | 18* | Number of overdoses in the past three months *(non-validated item added in 2021)* | Numeric (0, 1, 2–5, 6–10, >10) | Descriptive Only (not scored) |

**Appendix A2. Brief Addiction Monitor (BAM) Domains, Items, and Scoring Overview**
