# Supplementary material for: Tracking functional recovery in a community-based substance use disorder program: a five-year descriptive evaluation using the brief addiction monitor
Source: Addict Sci Clin Pract. 2025 Dec 6;21:4. doi: 10.1186/s13722-025-00625-3 (PMC12805790; doi:10.1186/s13722-025-00625-3)
Supplement: Supplementary file 2 — Supplementary Material 2 [file 13722_2025_625_MOESM2_ESM.docx]

| **Number of BAMs Completed** | **Time Interval of BAM Completion (in Months)*** | | | | | | | |
| --- | --- | --- | --- | --- | --- | --- | --- | --- |
|  | **3 months** | **6 months** | **12 months** | **24 months** | **36**  **months** | **48**  **months** | **60 months** | **>5 years** |
| 0 | 1,741 | 2,120 | 2,088 | 2,197 | 2,331 | 2,388 | 2,414 | 2,416 |
| 1 | 591 | 266 | 205 | 80 | 60 | 20 | 5 | 5 |
| 2 | 82 | 34 | 106 | 72 | 15 | 7 | 6 | 3 |
| 3 | 10 | 5 | 24 | 40 | 11 | 5 | 0 | 1 |
| 4 | 1 | 0 | 1 | 23 | 4 | 5 | 0 | 0 |
| 5 | 0 | 0 | 1 | 9 | 4 | 0 | 0 | 0 |
| 6 | 0 | 0 | 0 | 4 | 0 | 0 | 0 | 0 |
| *The months refer to the interval since each BAM assessment, not time since initial program enrollment. BAMs were intended to be administered at 90-day intervals, though actual timing varied. | | | | | | | | |

**Appendix Table A1. Distribution of BAM Assessments Completed by Time Interval Since Assessment (Not Enrollment)**
